# Delivery of alcohol brief interventions in community-based youth work settings: exploring feasibility and acceptability in a qualitative study

**DOI:** 10.1186/s12889-017-4256-1

**Published:** 2017-04-24

**Authors:** Martine Stead, Tessa Parkes, Avril Nicoll, Sarah Wilson, Cheryl Burgess, Douglas Eadie, Niamh Fitzgerald, Jennifer McKell, Garth Reid, Ruth Jepson, John McAteer, Linda Bauld

**Affiliations:** 1 Institute for Social Marketing, Faculty of Health Sciences and Sport, University of Stirling, Stirling, FK9 4LA UK; 2 Faculty of Health Sciences and Sport, University of Stirling, Stirling, FK9 4LA UK; 3 Nursing, Midwifery and Allied Health Professions Research Unit, Faculty of Health Sciences and Sport, University of Stirling, Stirling, FK9 4LA UK; 4 Faculty of Social Science, Colin Bell Building, University of Stirling, Stirling, FK9 4LA UK; 5 Institute for Social Marketing, UK Centre for Tobacco and Alcohol Studies, Faculty of Health Sciences and Sport, University of Stirling, Stirling, FK9 4LA UK; 6 NHS Health Scotland, Edinburgh, UK; 7 The Scottish Collaboration for Public Health Research (SCPHRP), 20 West Richmond Street, Edinburgh, EH8 9DX UK

**Keywords:** Alcohol, Brief intervention, Implementation, Young people, Youth work, Brief advice, Qualitative research, Feasibility, Acceptability

## Abstract

**Background:**

Alcohol Brief Interventions (ABIs) are increasingly being delivered in community-based youth work settings. However, little attention has been paid to how they are being implemented in such settings, or to their feasibility and acceptability for practitioners or young people. The aim of this qualitative study was to explore the context, feasibility and acceptability of ABI delivery in youth work projects across Scotland.

**Methods:**

Individual, paired and group interviews were conducted with practitioners and young people in nine community projects that were either involved in the delivery of ABIs or were considering doing so in the near future. A thematic analysis approach was used to analyse data.

**Results:**

ABIs were delivered in a diverse range of youth work settings including the side of football pitches, on the streets as part of outreach activities, and in sexual health drop-in centres for young people. ABI delivery differed in a number of important ways from delivery in other health settings such as primary care, particularly in being largely opportunistic and flexible in nature. ABIs were adapted by staff in line with the ethos of their project and their own roles, and to avoid jeopardising their relationships with young people. Young people reacted positively to the idea of having conversations about alcohol with youth project workers, but confirmed practitioners’ views about the importance of these conversations taking place in the context of an existing trusting relationship.

**Conclusion:**

ABIs were feasible in a range of youth work settings with some adaptation. Acceptability to staff was strongly influenced by perceived benefits, and the extent to which ABIs fitted with their project’s ethos. Young people were largely comfortable with such conversations. Future implementation efforts should be based on detailed consideration of current practice and contexts. Flexible models of delivery, where professional judgement can be exercised over defined but adaptable content, may be better appreciated by staff and encourage further development of ABI activity.

## Background

The World Health Organization (WHO) has defined alcohol brief interventions (known as ABIs in Scotland but also as SBI – screening and brief intervention - and SBIRT – screening, brief intervention and referral to treatment) as ‘practices that aim to identify a real or potential alcohol problem and motivate an individual to do something about it’ ([1], p6). The Scottish Government defines an ABI as:

*‘… a short, evidence-based, structured conversation about alcohol consumption with a patient/client that seeks in a non-confrontational way to motivate and support the individual to think about and/or plan a change in their drinking behaviour in order to reduce their consumption and/or their risk of harm’* ([2], p2–3).

Often considered a family of interventions, ABIs are heterogeneous and vary in length, content, delivery, deliverer and target group [3]. Despite this they have a structure and style that distinguishes them from simply advising a person to drink less. Key components include obtaining an accurate picture of a person’s alcohol consumption, a collaborative style of conversation, and use of techniques to support health behaviour change. The early history of development of ABIs was based on the concept of behaviour change as a process rather than an event [4], informed by the transtheoretical model of change [5]. In more recent years, ABIs have continued to vary, and include those based on simple advice [6], those based on the style of motivational interviewing [7], and those involving core skills of motivational interviewing [8, 9]. The study of mechanisms of action of ABIs in order to identify necessary components is at an early stage [10, 11].

While there is robust evidence supporting the efficacy of ABIs in reducing alcohol consumption in adult hazardous and harmful drinkers in primary care settings [12–14], a series of reviews has shown that evidence for the efficacy of ABIs with young people is inconclusive [15–17]. In addition, a review of reviews of ABIs for adolescents [16] concluded that no recommendations could be made on ‘target population, setting, screening tool or intervention approach’, and that further research was needed to develop screening tools with incremental age-appropriate cut-offs for identification of alcohol problems. Overall there is limited literature exploring ABIs with young people in settings other than formal health and education, and few studies from outside the USA [18].

Despite the lack of research evidence for the effectiveness of ABIs with young people, there is policy support for these interventions because of the risks and harms connected to harmful and hazardous use of alcohol by young people [19]. In Scotland, ABIs are a policy priority as part of a broad-based alcohol strategy aimed at addressing high levels of harm related to alcohol. In 2007 the Scottish Government set a target for ABIs to be delivered by NHS Health Boards between April 2008 and March 2011 [20] across three settings: primary care, accident and emergency, and antenatal care. In 2012 the target was revised so that up to 10% [21], and later 20% [22], of ABI delivery could be achieved in other settings. A Scottish scoping study [23] found several instances of local implementation of ABIs targeting young people in settings outside of formal education, often in community services for young people. In light of such implementation in Scotland, and the limited scrutiny of feasibility and acceptability of ABIs in these settings, the study reported here aimed to explore the context and approach, and feasibility and acceptability of ABI delivery in youth work projects across Scotland. The two central research questions informing this study were: how were ABIs delivered in these settings, and how feasible and acceptable were the ABIs to staff and young people?

## Methods

### Sampling and recruitment

Twelve project contacts were identified from an initial scoping exercise conducted by the project funder [23]. The academics who undertook the research were provided with contact details for all of the projects which responded, all of whom consented for their details to be shared. A further project was identified by the research team and contacted during the recruitment phase. All nine projects that confirmed they were either delivering ABIs to young people or considering doing so in the near future consented to take part in the study, ensuring maximum variability was achieved from responders. From this sample of nine projects a practitioner sample was constructed comprising 21 project managers, staff and related stakeholders, such as those providing training to the project or working in partnership with it, identified through purposive sampling in order to access a diversity of opinions. Young people were recruited from six of these nine projects. These six projects were actively working with young people on alcohol (including delivering ABIs) at the time of study, and agreed to allow the research team to contact their service users. Access to the young people (*n* = 61) was negotiated individually with project staff. Sample stratification was not possible due to the unpredictable nature of those accessing the projects. Efforts were made, however, to include both females and males and a range of ages and numbers sufficient to achieve data saturation. Fieldwork was conducted in two phases (staff followed by young people) between December 2012 and September 2013.

### Data collection

Semi-structured in-depth interviews were conducted with practitioners (*n* = 21) to explore in detail their experiences, reasoning behind adoption of ABIs, and practices. Interviews were considered the best method to carefully examine staff perceptions and the processes of ABI delivery within the projects. A mixture of face-to-face and telephone interviewing was used and interviews lasted between 24 and 100 min. Questions explored included: the project history/setting and host organisation; overall project target population/client group and size; staff training in relation to alcohol; goals of ABI delivery and intended outcomes; context and manner in which ABIs were being delivered; use and perceptions of screening tools; and perceptions of how young people responded to ABIs. Time was provided for participants to raise issues of importance to them. Relevant project documentation was also collected, including project screening instruments, activity monitoring forms, and internal reports offered by participants, supplemented by any publicly available information from the internet. Where possible, observational field notes were also collected. All staff participants were forwarded a copy of the project information sheet and consent form by email in advance of the interview in order to request informed consent. For face-to-face interviews, participants were asked to sign a copy of the consent form at interview, and for telephone interviews consent was indicated by email.

Young people (*n* = 61, 37 male and 24 female participants with an age range of 12–23 years) were interviewed in focus groups and paired interviews which created a convenient way to talk with the young people in a natural way that was considered to enhance validity and where some of the group dynamics at work in these settings could also be observed. Interviews lasted between 15 and 45 min (some constrained by cold outdoor conditions on the side of football pitches) and focus groups ranged between 15 and 90 min. Young people were given a gift voucher to acknowledge their participation. Questions explored young people’s perceptions and experiences of the project, such as practical aspects and perceptions of staff approachability, trustworthiness, credibility and communication style; recall of having had a conversation about alcohol at the project and the nature of that conversation; participants’ willingness to be asked questions about, and comfort with, talking about alcohol; perceptions of the relevance, usefulness and feasibility of the advice given and whether participants had acted on it or made any changes in response to the ABI. Interview topic guides were adapted to take into account young people’s familiarity with and experience of ABIs. If they were not familiar with the concept, they were asked how they felt about having conversations with project workers about alcohol and their own drinking. Observational field notes were also collected. The interviews with young people were conducted by research team members with extensive experience of conducting research interviews with children and young people. One was a qualified social worker, and all had obtained relevant certification for working with young people. Training was provided on the specific subject of ABIs, and supervision was provided by senior members of the team, who also ensured that research interviews met the required standard. In most cases, two research team members were present at interviews which helped to ensure consistency and quality control.

Young people were informed about the research in various ways. Many were given information leaflets about the study by project staff and the research process explained to them in advance of the interviews. Others, such as those attending sports-based projects, were informed about the research on the evening that the interviews took place. All young people were given a study information sheet. Parental consent was not sought, as this was felt to be inappropriate given that young people were accessing projects relating to alcohol use and sexual health, about which their parents may not have been aware. Instead, project staff assessed the capability of each to make an informed decision about their participation in the research. At the beginning of the focus group or interview, the research was explained to young people in clear and jargon-free language. They were asked to complete a consent form which clearly stated that they understood the research, were given the opportunity to ask questions about it and were reassured that they were free to withdraw from participation at any stage of the interview.

Where permission was given, interviews and focus groups with staff and young people were recorded on digital audio file (one interview was recorded using hand-written notes due to battery failure), and transcribed verbatim by a professional transcriber. Twenty young people did not wish their interviews to be recorded and these were documented in detail using interview case notes. Participants’ anonymity and confidentiality were ensured.

### Analysis

Interview data with practitioners and project observation notes were uploaded into the NVivo data analysis computer software programme. A thematic analysis drawing on the Framework [24] approach was used for the coding, categorising, summarising and analysis of data. Data analysis was closely guided by the research questions and objectives but also allowed for open coding. Two researchers (AN and TP) coded the staff interviews and field visit data, with regular discussions taking place throughout regarding coding decisions and coding labels for themes where there was uncertainty. The documentary sources were used alongside primary data to construct anonymised project case summaries that were checked with project staff for accuracy. Members of the broader research team were involved in commenting on the emerging analysis and write-up of findings. For the young people interviews a thematic approach was also used where transcripts (or interview notes if permissions were not provided) and field notes were analysed independently by the two researchers involved in the young people’s data collection (SW and CB). Again, analysis was guided by the research questions and study objectives, in addition to use of open coding to incorporate emerging themes. In reporting study findings anonymised quotes are used, and we note whether the interviewee had a front line or strategic view on delivery and whether the interview was individual or paired. For the individual interviews with young people, gender and age are provided.

### Ethical issues

Ethical approval for the study was provided by the University of Stirling Management School Research Ethics Committee. Consultation with local NHS Research Ethics Services indicated the study did not require NHS ethical approval. None of the researchers held other roles within the projects.

## Results

In this section we provide an overview of the aims and approaches to ABI delivery across all of the projects, the factors affecting feasibility of the projects and the acceptability of ABIs for both staff and young people.

### Project aims and approaches

The nine included projects were heterogeneous in terms of settings, target groups, staffing and intervention approach, as shown in Table 1, in which pseudonyms have been used to protect the confidentiality of the projects. Data collection allowed a ‘snapshot’ of projects that were in different stages of development.

**Table 1:** Project approaches to ABI delivery

Project name	Type of setting(s)	Target group age	Activities	Time	Agencies	ABI service	Screening tool
Aspen	Community diversionary (sport)	15–17 years	Football coaching	Friday evenings	NHS health improvement team, youth provider (football coaching), voluntary sector alcohol organisation, police	Football coaches offer screening and basic feedback with registration	CRAFFT
Bracken	Mobile outreach	14–16 years	Team of 4 (youth worker, nurse, police officer, detached youth worker) on customised bus driven to young people, usually following intelligence and referrals	Friday evenings	Voluntary sector substances misuse and information service for young people, NHS health improvement, community learning and development detached youth team, police	Screening and ABIs offered by youth worker or nurse on board bus. Includes card game to structure thinking. Individual or paired. Less structured conversations take place outside the bus. Follow-up at school by youth worker.	CRAFFT
Elder	Community diversionary (sport)	10–18 years	Indoor and outdoor sports coaching	Weekday evenings	Local authority leisure services (sports development), NHS (training and support)	Opportunistic conversations about alcohol with coaches at side of pitch	Not used
Fir	Hybrid (centre based; outreach; streetwork; diversionary)	12–21 years	Open access drop-in, group activities, appointment-based support and counselling, schools, youth clubs and facilities, streetwork, sport, outdoor activities, wilderness residential experiences	Flexible	Confidential health and well-being centre and service for young people, streetwork team, community youth organisations	ABIs offered as appropriate by Fir staff (youth workers). Individuals or small groups.	CRAFFT
Hawthorn	Pilot project – training delivered	Not clear	Training targeted at organisations working with young people who don’t access traditional services	Not known	Training by Alcohol & Drugs Partnership and national workforce development organisation. Implementation by football coaches, council and voluntary sector youth workers, NHS health improvement and psychological therapies staff.	Not known	CRAFFT
Juniper	Police-referred	12–18 years	Young people identified through routine police work	Negotiated	Third sector children’s organisation, police, Alcohol & Drugs Partnership (funder)	Project arranged to see young person at home/place of choice for ABI. Telephone follow-up after 3 months.	Customised
Myrtle	Hybrid (centre-based; outreach; streetwork; diversionary)	11–17 years	Mentoring, intensive support, drug and alcohol outreach, streetwork, mobile street football	Flexible	Voluntary sector social services organisation, Alcohol & Drugs Partnership, police, social work, education, community learning and development, street pastors	Offered by Myrtle staff as a normalised part of practice to introduce young people to the service and structure decision-making	Approach taken from national policy guidance document [2]
Pine	Drop-in centre	13–18 years	Drop in centre (sexual health, housing, support, employment or relationship concerns, substance use)	6 days a week	Confidential health, information and peer-led service for young people, NHS, local authority	ABIs offered as appropriate by staff (sexual health nurses, community learning and development worker, health promotion workers)	CRAFFT
Rowan	Police-referred	12–17 years	Young people identified through intelligence-led targeted police campaigns and brought to the police station. Parents are called in if <16.	Friday evenings	Police, voluntary sector youth project, voluntary sector drug and alcohol service	Young people introduced to the team, then ABI offered by voluntary sector organisation the following week	FAST

As shown in Table 1, the projects operated in a range of community settings including community centres which young people visited for advice and support, mobile drop-in vans which visited communities, the side of sports pitches, and street outreach. In addition, some projects worked primarily through one approach, such as provision of diversionary activities, while others used a variety of linked approaches. Some projects used relatively structured approaches such as sexual health appointments, where ABIs could be delivered in similar ways. Others worked in a much less formal way, for example with conversations about alcohol taking place opportunistically at the side of a football pitch. One project (Hawthorn) was very early in the process of considering adoption of ABIs, so limited information was available at the time of data collection.

Various drivers of alcohol work were identified, including as an early intervention health promotion strategy that would benefit young people later in life, particularly against a backdrop of Scotland’s culture of normalised alcohol use. Other drivers were to address the negative impact of alcohol on young people’s lives (including injury, the consequences of unprotected sex, domestic violence, and anti-social behaviour); to engage with vulnerable young people around crime and anti-social behaviour issues; and to provide alternative activities to drinking alcohol, such as sport. These drivers were responsible for the very different approaches that developed within each of the projects.

ABIs, as adapted by the projects, were generally seen as one component of the work that staff needed in order to address alcohol issues in their service or setting, rather than the only approach. ABIs were described as a *way of working* with young people, rather than as a one-off discrete intervention, and as part of a process taking place over several sessions to engage, build up trust and encourage young people to a stage of readiness for more extended work such as motivational interviewing.

### Factors affecting the feasibility of ABIs in youth work settings

Practitioners identified organisational funding arrangements and financial stability as important factors in terms of the longer-term feasibility of delivery of ABIs in youth work settings. The projects varied widely in terms of their financial security, with some having been in operation for 30 years or so and others operating on a more precarious basis. One project came to an end during the study because of a lack of future funding and another reported 12 different funding sources. For some there were no additional funds for ABI work. Funding issues affected ABI delivery in several ways: limited resources and insecure funding affected the priority given to ABI work, particularly if ABIs were seen as an ‘add-on’ to core activity; training for ABI delivery was not necessarily seen as a worthwhile investment where project continuation was uncertain; and longer term follow-up of those who receive ABIs was less viable if a project was dependent on short-term funding contracts. On the other hand, where ABI work was used in some cases to support a case for additional funding, this could be a driver of ABI activity:

*‘The term ABI is really useful for us because we can package that when we are speaking about alcohol into something that other people understand, funders understand, so in some ways it’s giving us credit for the work that is going on but in other ways funders really like that because they can understand it a bit more and so they push for it’* (Practitioner, Individual interview).

Practitioners reported varying levels of confidence, training and experience in delivering ABIs. Many of the teams were multi-agency and multi-disciplinary, and this diversity was felt to be important for ABI delivery, particularly where teams included health professionals with expertise on alcohol and its relationship to other health issues, and knowledge of specialist services.

Practitioners commented that the location, timing and targeting of ABI delivery, along with other services relating to alcohol, needed to acknowledge and adapt to changing patterns of local drinking where necessary, and to adopt a tailored approach to different sub-populations where appropriate. Where projects were engaged in outreach, a key challenge was knowing where young people were going to be involved in street drinking and therefore where to take the outreach vehicle or activity. A number of participants stated that street drinking levels seemed to be decreasing during the time of the study, with some interviewees suggesting that parents were supplying alcohol at home as it became harder for young people to buy alcohol in commercial outlets due to, for example, community off-sales campaigns. This change in local patterns of drinking was clearly impacting on projects set up to address street drinking by young people:

*‘We make assumptions that because young people have this pattern just now that in six months or a year’s time that that will still be their pattern and that’s not always the case. It may well have been a perceived success in that they moved the young people off the streets so that they weren’t getting the calls about the young people. But everybody including the police are well aware that these young people haven’t stopped drinking. And in many ways when they are drinking inside and they are not visible they may be more vulnerable and more at risk’* (Practitioner, Individual interview).

The quote here indicates the changing nature of vulnerability based on the patterns of young people’s drinking behaviour. The implication for feasibility is that projects realised they would only be successful in working proactively with young people and addressing such vulnerability, including through the use of ABIs, if they were extremely flexible, ‘nimble’, and responsive, able to change their style, and even place, of working in response to shifts in young people’s behaviour in their area.

### Acceptability of ABIs to staff and young people in youth work settings

Project staff highlighted particular *dimensions of engagement* as essential when delivering ABIs to young people and these had clear implications for the acceptability of ABIs in such settings. Two intrinsically-related dimensions were the importance of being flexible, responsive and opportunistic, and creating trusting, respectful and non-judgemental relationships. These two themes are now described in turn.

#### Being flexible, responsive and opportunistic

Where a project’s delivery approach included streetwork models such as mobile vans visiting communities that young people viewed as their ‘territory’, practitioners made great efforts to find out where young people were meeting, rather than expecting young people to come to them. This necessitated keeping abreast of changes in local drinking patterns and drinking locations in order to be able to find young people with whom to work. ABI delivery then tended to be opportunistic, with conversations about alcohol often initiated by young people themselves.

Even in non-mobile youth settings, flexibility was a key feature of the delivery approach and practitioners had to be ready to offer an ABI if and when an appropriate situation arose:

*‘I wouldn’t do it* (an ABI) *formally every single time with every young person. It would depend on what they have disclosed through the consultation. You know, things like if someone has come in for emergency contraception, when did it happen, or if they came in for a pregnancy test, when did it happen? “OK, so why did you not use a condom that night?” “I was drunk”. Alright, so that is an ‘in’ for me, how drunk were they? Were they able to give consent? Who bought the alcohol? There is a whole load of discussions around that kind of thing, about the risk taking behaviour and that’s the one that I might go on and use the CRAFFT tool for’* (Practitioner, Individual interview).

A number of comments were made about the importance of employing open-ended questions and a friendly, conversational and informal style, to help a young person explore their alcohol use and come up with their own solutions. Furthermore, staff viewed ABIs as opportunities to use ‘regretted behaviour’, rather than traditional health messages, as a lever for change:

*‘If you are working with a 16 year old, well they are invincible. They are unlikely to be experiencing any health-related issues there and then and you can say " well you can be storing up some health problems for when you are 35 or 40", [but] that’s a lifetime away for them. So what we have tended to focus on is what we refer to as regretted behaviour. And that feels as though it’s much more powerful to young people' (Practitioner, Individual interview).*

Use of ABIs was often driven by an ethos of harm reduction: staff accepted that young people would engage in risk-taking behaviours, so aimed to help them minimise risk, rather than eliminate it, through exploring strategies such as drinking water, eating, getting home safely and looking after friends. In some cases, where police or criminal justice staff were involved, these behaviour changes were not always viewed primarily in terms of improving health but rather as a means to minimise social disorder. Participants believed that this, in turn, could have an impact on how the success of an ABI delivery initiative was perceived (e.g. in terms of fewer reports of young people creating disorder on the street, rather than reductions in drinking per se). In other words, broader behaviour change was viewed as relevant to these initiatives as well as reduction of alcohol consumption and harms. In this respect, a number of staff believed that their delivery of ABIs was having a positive impact:

*‘We’ve also had a lot of young people when we’ve done the follow-ups that have said that the ABI was enough to make them think about their alcohol. So if I hadn’t had that conversation that night… they’ve not needed any more support after the ABI. It’s not just about their alcohol intake, it’s about their safety and changes in their behaviour’* (Practitioner, Individual interview).

Practitioners emphasised that it often took a long time to build up confidence with young people, especially in streetwork. They believed young people lacked trust in professionals who went through ‘tick-box’ lists or had short, fixed-time appointments, so attempted to give control back by keeping the young person informed about the necessary processes:

*‘All the paperwork I use I explain to the young people what the paperwork is and why I am using it. That’s just my personal preference because I think that sometimes professionals sit there and fill out things and type things out on computers and people sit and go" what are they saying about me?"﻿. Especially young people " am I being judged? What’s my name going down on?". So if you explain to them what it is, then that gives them that element of control back’* (Practitioner, Individual interview).

These issues concerning the use of paperwork with young people had implications for ABIs where assessment tools were usually considered intrinsic to the consultation. In some of the projects we investigated, screening was not part of routine practice because it was considered to be off-putting to young people. Practitioners spoke about focusing on the advisory and educational aspect of the ABI, rather than on formal screening, which sometimes appeared to be overlooked or to go unrecorded. Other projects did report using screening tools, such as when registering for an activity, and had found ways to make such tools acceptable to young people. However, some project workers felt there was a lack of suitable alcohol screening tools for use with young people, and had adapted tools to provide a better ‘fit’ for their projects. Examples of adaptations made by the Fir project included modifying the language (for example, not referring to units when discussing how much alcohol the person consumed), and focusing on regretted behaviour and harm reduction rather than discussing long-term health consequences. If screening took place, the CRAFFT tool was used, modified in a credit card-sized version and asking only about alcohol. Population screening of all consenting clients over a month had been carried out twice, 6 months apart, with ABIs offered following a positive screen. Generally CRAFFT was popular with projects that had adopted a screening tool.

Overall, the structured framework of an ABI was generally viewed as having benefits for the youth workers because it created space to involve the young people in the process, helping to structure and organise their thinking about their drinking behaviour. At the same time, the ability to adapt the ABI, and make it less formal, was also important, particularly in terms of preserving the important practitioner-service-user relationship. Given that there was a perception among some project staff that an inflexible model of ABI delivery designed for adult and health care settings was sometimes being inappropriately expected of youth services, allowing staff teams to adapt the ABI process was key in them accepting this new way of working and making it their own.

#### Creating trusting, respectful and non-judgemental relationships

The principles of youth work, described by staff as starting with the young person’s view of the world and seeking to develop skills and attitudes rather than remedy problem behaviours, informed all decisions and activity in these settings, including delivery of ABIs. Non-judgmental engagement on young people’s terms was viewed as absolutely key. The work with young people placed a strong emphasis on the relationship between the worker and the young person. This included developing the trust on which a relationship can be built, maintaining it, and avoiding or limiting actions or events that might jeopardise the relationship. It was important for staff to work intuitively to build up rapport and make the contact fun and relaxed for the young person, as this worker describes:

*‘Normally it would be somebody that you’ve seen a couple of times, you wait until they come to the point where they are quite open and chatty about their life [or] at least receptive to talking about themselves and talking about their own experience and then we bring it up. So either you* *know* *an experience has happened that they want to talk about or reflect on, or you* *know* *they are just talking about generally using alcohol quite a bit and so OK, let’s work out how much you are drinking’* (Practitioner, Individual interview).

Some practitioners expressed concern that ABI work might bring an unwanted power inequality to this relationship, fearing that ABIs, if not adapted to some degree, were too ‘clinical’.

*‘We are in the youth work game. We’ve got… we build up rapport, relationships. They know that we care and if they know that we care then they are willing to come with us. But we are going to have to look at the balance between, is the ABI something that is quite clinically done, or is there the opportunity to bend it a little to make it fit with youth work methodologies and models?’* (Practitioner, Individual interview).

Not all the young people interviewed had experienced an ABI per se*,* although they recalled conversations about alcohol, and some were unsure whether they had had an ABI or not.

Young people’s response to the idea of having conversations about alcohol with project staff was strongly bound up with their wider feelings about the projects and workers. The young people interviewed were mostly very positive about the projects themselves, perceiving them as welcoming and safe places, within broader environments that were not generally perceived as so supportive. This attitude towards youth projects contrasted with some participants’ perceptions of other agencies including health care services, which they tended to avoid, and school authorities, which could not ensure confidentiality. Several young women who attended a health project explained their reluctance to spend time outside, including in parks, as a consequence of constant police attention. One described the local area as ‘a ghetto’; her friend further explained that attending the project ‘*gets you off the streets and out of trouble’* (Young woman, aged 14).

Young people’s views of youth work staff were generally very positive, pointing to high levels of trust. As one young man (aged 18) explained:

*‘Youth workers are more like your pals - they have a laugh with you. You can tell them what you were doing at the weekend and they don’t shout at you, they find other ways to speak to you’.*

Overall, young people felt valued by staff in a way that reflected more respectful relationships than those experienced with other adults they encountered. In this context, staff members were valued as credible and confidential/trustworthy sources of advice and support:

*‘It’s good because you’ve got someone to speak to other than your pals. I can tell ‘P’ about anything and it won’t go any further. She’ll give you sensible advice rather than you getting advice off your pals - friends’ll say just drink and P’ll say you’ve got to think about it*’ (Young woman, aged 16).

*‘They* [the youth workers] *have the right approach - they don’t threaten you with the police or give you a lecture or use scare tactics. They give you advice’* (Young man, aged 17).

As such, the young people interviewed were largely amenable to conversations about alcohol, or to the concept of conversations about alcohol, and felt that these fitted with the perceived concern that youth workers had for their wellbeing. It is worth noting that, although some practitioners expressed concerns about potentially jeopardising relationships with young people by introducing the topic of alcohol, these did not appear to be borne out by the interviews with young people themselves, who seemed largely open to the concept of talking about their alcohol use with staff, providing they knew and trusted those staff. At the same time, they disliked form-filling and the sudden introduction of activities which felt formal and official, and appreciated efforts to make conversations about alcohol more engaging and less formal:

*‘I think they approach it in the best way because it’s informal and they do it with activities and games and talking’* (Young woman, aged 15).

*‘Activities are good - they help more than boring facts’* (Young man, aged 17).

Some who had experienced ABIs, or similar conversations, welcomed the one-to-one format. They felt that it enabled them to gain a more accurate sense of how much they were drinking at different times, and to be more honest about their alcohol use:

*‘There was a sort of screening and they brought it up, but not too ‘in your face’, not in that sort of way. Think it was an ABI – it lasted 10-20 minutes and then it was a group discussion after. The ABI bit was important to do on your own as it’s confidential and you might not be so honest in a group’ (Young man, aged 17).*

In summary, ABIs were seen as offering a useful approach to talking about alcohol, by both practitioners and young people. Such conversations were deemed important to have in youth work settings because of alcohol’s role in many of the health and social issues affecting young people.

## Discussion

The study findings suggest that ABIs are both feasible and acceptable in community youth work settings. Practitioners perceived that there was value and benefit in speaking with young people about alcohol in ways that fitted with the broad definition of ABIs. However, importantly, ABIs were perceived less as a discrete intervention and more as a way of working with young people and part of the process of developing young people’s readiness to engage in more extensive work around alcohol. Young people were generally positive about the idea of having conversations with project staff about alcohol, providing that they knew and trusted the staff involved, though they did not always recognise such conversations as ‘ABIs’. Several of the young people interviewed reported avoiding health services where possible, and being mistrustful of other agencies, making ABI delivery in youth work settings an important mechanism to reach such individuals.

Our findings have outlined how the delivery of ABIs to young people differed in several key ways from typical conceptualisations of ABIs in mainstream health settings, such as primary care, particularly in their more opportunistic nature and more relaxed approach to use of structured tools and form-filling. These differences arise from the wider context in which ABI work is situated in the youth work setting, in the ethos and values of work in this setting, and in the perceived needs of young people receiving ABIs compared with adults. A particularly important point to emphasise is that there was no single homogenous ‘youth setting’, but rather a wide heterogeneity of settings, some of which shared common features while others were very different. Organisational funding arrangements and financial stability in particular were important factors in terms of the longer-term feasibility of delivery of ABIs in youth work settings.

One key difference between the health services in which ABIs are generally delivered with adults and some of the settings where ABIs were delivered in this study related to the nature of clients’ attendance at services. Unlike in primary health care, the young people in our study often attended projects on a ‘one off’ or drop-in basis. The often unscheduled and unpredictable nature of such attendance raised particular challenges for structured follow-up, something that is viewed as good practice in ABI delivery as well as in tracking the effectiveness of an intervention in terms of outcomes. While these challenges can also be present in health settings, such as accident and emergency departments [20], they are arguably more difficult to overcome in youth settings than in typical adult health services.

The study has raised questions regarding what an ABI means when delivered in such flexible and diverse ways as found in this study. Heterogeneity seems a particular feature of ABI delivery in youth settings but is not unique to them. As noted earlier, the wider research literature indicates that ABIs are heterogeneous and vary in ways that are not always clearly described [3]. Studies have noted a range of challenges relating to, for example, the consistency of the ABI process [25]; the ability of practitioners to control the intervention [26, 27]; privacy concerns [26, 28, 29]; the need for flexibility on the part of the worker delivering the ABI [30, 31]; and potential difficulty in formalising and recording the intervention [27, 30, 32]. The need for further study and understanding of the content of ABIs has also been noted [11], as well as the need to be clearer about core and adaptable components in this kind of intervention [10, 33]. Avoiding overly rigid conceptualisations of ABIs has been found to be important in other settings outside of traditional primary care [30, 32]. Practitioners clearly see the need for different varieties of intervention that can meet young people’s needs, but so far there has been limited progress on how ABIs should be designed for settings such as these where alcohol problems frequently present [32, 34, 35].

In this study ABI delivery tended to be opportunistic, with conversations about alcohol initiated more frequently by the young people themselves than would be the case in mainstream health services. In some other settings, guidance is available to staff on when to raise alcohol in a way that relates it to a client’s presenting issue (e.g. the 10 presenting signs of the Paddington Alcohol Test designed for A&E settings [36]). These settings are also expected to screen and/or deliver ABIs opportunistically based on each individual presentation. It is therefore conceivable that similar guidance on presenting issues could be developed specific to different youth projects or services, and drawing on youth work experience.

The acceptability of ABIs to workers in youth work settings was strongly influenced by their perceptions of the value and benefits of ABIs, and the extent to which these interventions were perceived to fit coherently with the aims and ethos of the projects’ wider context of work with young people. ABIs were feasible and acceptable to youth workers where they could be incorporated into practice in ways that did not threaten working relationships with young people. This concern for preserving the client relationship when delivering ABIs is not unique to youth work. Studies have found that both antenatal settings [37] and primary health care settings [38, 39] place importance on nurturing and maintaining the relationship with clients in ABI delivery. It is therefore likely that judgements about how ABIs might affect practitioner-service user relationships will be a primary concern shaping whether and how ABIs are used across a range of settings. One of the implications of our study is that those designing ABIs and ABI training need to design ABIs in ways that understand the importance of this relationship. Standardised and inflexible approaches to ABI delivery would not be seen as appropriate or adopted uncritically in settings such as the ones we studied. Rather, our findings have shown that ABIs will be more likely both to be valued as an *approach* (rather than an intervention), and to be *adopted*, if there is flexibility created for their implementation. In other words, they will be more accessible to these types of settings if they are viewed as part of a repertoire of approaches that workers can draw on, as and when needed and circumstances allow. Indeed, the ability to ‘dip in and out’ of different approaches to communicating with clients is an important skill for proficient use of a motivational style of working with clients [8] and inherent in ABIs as defined in Scotland [7].

Interestingly, although practitioners were concerned about potentially jeopardising relationships with young people by talking about alcohol, young people themselves appeared fairly comfortable with such conversations. A similar apparent mismatch in perceptions has been found in primary care, whereby practitioner concerns about alcohol discussions causing offence to patients are not always shared by patients themselves [40–43]. It should be emphasised, however, that in this study young people’s apparent comfort with the concept of alcohol discussions reflected the very positive relationships which they reported having with the project workers, and the time and effort put in by these workers to build trust. In other words, it should not be assumed that young people would be equally accepting of ABIs delivered in other more formal contexts or by other workers where these positive foundations were not in place.

There was a perception among some project staff that an inflexible model of ABI delivery designed for primary health care settings was sometimes being inappropriately expected of youth services. Similar criticisms were made of the Scottish ABI programme in a previous study in A&E and antenatal settings in Scotland [30]. Difficulties with recording screening and brief intervention delivery were also documented in that and other studies in A&E [27] and other healthcare settings [44]. The problem of accurately recording screening and brief interventions also figured as a key finding in the national evaluation of ABI implementation across all three settings of primary care, A&E and antenatal settings [20]. In our study a clear need was identified for screening or assessment methods and resources that would be suitable for ABIs with young people, and the lack of such resources reflects the still developing nature of ABI work in youth work and other settings. This is an area where future research and development should be directed.

### Research and policy implications

Whilst ABIs appear to be feasible and acceptable in this setting, it is unlikely that all forms of ABI currently being delivered are equally effective. It is not impossible that some may be counter-productive [45]. Further research is therefore critical prior to largescale implementation initiatives [46]. Such studies will face several challenges, given the ways in which ABI intervention components were negotiated in practice by the projects including appropriate intervention design, fidelity to the intervention under study, and minimising research participation effects [47]. Traditional randomised controlled trial designs “may not be best suited for evaluating complex interventions in dynamic environments” [48] like this and researchers may wish to consider alternative trial structures such as Solomon four-group designs to address screening and other research participation effects [49, 50]. These have ethical implications that require further debate [51]. Other non-randomised designs such as ‘stepped wedge’ may also be valuable [52]. In the meantime, practitioners in this field need clear guidance and policy on how best to discuss alcohol with young people based on current evidence, notwithstanding the strong evidence base favouring legislative rather than individual interventions [53].

### Strengths and limitations of the study

The ABI activity explored was not set up for the purposes of this study but had evolved naturally in various settings in Scotland, in part driven by the growing profile of ABI delivery in adult health care settings. Consequently, the study provides a detailed insight into the realities and challenges of ABIs in real world settings, and into the perceived acceptability, feasibility and value of ABIs for young people and for the staff in these settings. The study was not set up to evaluate the impact of ABIs in the projects, something that would not have been possible without further work to develop record-keeping and follow-up systems and consideration of the challenges discussed above.

To our knowledge, this is the first time that ABIs delivered to young people in community (non-school) settings have been examined outside of the context of a research trial. A particular strength of the study lies in bringing together multiple perspectives and data sources on ABI delivery: it is relatively rare to hear the voices of those on the receiving end of ABIs, particularly young people. The study also has several limitations. It did not include every project in Scotland delivering ABIs to young people at the time, only those known to the study commissioners and the research team which then consented to take part. The young people interviewed were either selected by project staff or were self-selecting, and could therefore have been those more likely to be engaged with (and positively disposed towards) the projects. This might have influenced their response to ABI delivery. That said, many of the young people participating had not been pre-selected by projects but were simply those who happened to be present at the project, and willing to participate when researchers visited. For some young people interviewed, ABIs had little relevance because they did not drink. This is less a weakness of the study but instead provides insight into the importance of considering where ABIs might most usefully be targeted and delivered.

## Conclusion

This study found that ABIs are indeed feasible in youth work settings with some adaptation. Acceptability of ABIs by staff in these settings was strongly influenced by their perceived value and benefits, and the extent to which they were viewed as fitting coherently with the aims and ethos of the wider context of work. Although practitioners were concerned about jeopardising their relationships with young people by talking about alcohol, young people themselves appeared fairly comfortable with such conversations. In terms of implications for practice, our study findings suggest that future efforts to implement screening and ABIs should be based on detailed consideration of current practice in teams or settings targeted. Clear models of delivery should be developed with sufficient flexibility to allow staff to exercise professional judgement, while still having clearly defined content. As suggested by others [32], this would include adaptations to any existing assessments, documentation and recording procedures. The activities delivered in training could then focus on enabling staff to implement changes in practice to deliver ABIs in ways that had been agreed in advance. Such an approach is likely to be better appreciated by workers in these settings and to encourage the development of ABI activity [30, 54]. Future development, support and training will need to reflect the diversity and experience of delivery of ABIs in these settings.

## Abbreviations

CRAFFT: Car, Relax, Alone, Forget, Friends and Trouble [55]
FAST: Fast Alcohol Screening Test [56]
AUDIT: Alcohol Use Disorders Identification Test [57]

## References

[1] Babor TF, Higgins-Biddle JC (2001). Brief Intervention for Hazardous and Harmful Drinking. A Manual for Use in Primary Care.

[2] Scottish Government. Local Delivery Plan Standard: Alcohol Brief Interventions. National Guidance 2015–2016. 2015. http://www.show.scot.nhs.uk/wp-content/uploads/2015/06/Alcohol-Brief-Interventions-ABI-National-Guidance-2015-16.pdf. Accessed 14 Apr 2016.

[3] Heather N (1995). Interpreting the evidence on brief interventions for excessive drinkers: the need for caution. Alcohol Alcohol.

[4] McCambridge J, Cunningham JA. The early history of ideas on brief interventions for alcohol. Addiction. 2014;doi:10.1111/add.12458. 10.1111/add.12458 PMC3992901 24354855

[5] Prochaska JO, DiClemente CC. Stages and processes of self-change of smoking: toward an integrative model of change. J Consult Clin Psychol. 1983;doi:10.1037/0022-006X.51.3.390. 10.1037//0022-006x.51.3.390 6863699

[6] Kaner EFS, Bland M, Cassidy P, Coulton S, Dale V, Deluca P, et al. Effectiveness of screening and brief alcohol intervention in primary care (SIPS trial): pragmatic cluster randomised controlled trial. Br Med J (Clin Res Ed). 2013; doi:10.1136/bmj.e8501. 10.1136/bmj.e8501 PMC3541471 23303891

[7] Fitzgerald N, Winterbottom J (2009). Alcohol Brief Interventions Training Manual.

[8] Miller WR, Rollnick S (2012). Motivational Interviewing, Third Edition: Helping People Change.

[9] Zatzick D, Donovan DM, Jurkovich G, Gentilello L, Dunn C, Russo J, et al. Disseminating alcohol screening and brief intervention at trauma centers: a policy-relevant cluster randomized effectiveness trial. Addiction. 2014;doi:10.1111/add.12492. 10.1111/add.12492 PMC4014067 24450612

[10] Gaume J, McCambridge J, Bertholet N, Daeppen JB. Mechanisms of action of brief alcohol interventions remain largely unknown - a narrative review. Front Psychiatry. 2014;doi:10.3389/fpsyt.2014.00108. 10.3389/fpsyt.2014.00108 PMC4143721 25206342

[11] McCambridge J. Brief intervention content matters. Drug Alcohol Rev. 2013;doi:10.1111/dar.12044. 10.1111/dar.12044 PMC3746115 23819570

[12] Jonas DE, Garbutt JC, Amick HR, Brown JM, Brownley KA, Council CL, et al. Behavioral counseling after screening for alcohol misuse in primary care: a systematic review and meta-analysis for the U.S. Preventive Services Task Force. Ann Intern Med. 2012;doi:10.7326/0003-4819-157-9-201211060-00544.

[13] Kaner E, Dickinson H, Beyer F, Campbell F, Schlesinger F, Heather N, et al. Effectiveness of brief alcohol interventions in primary care (review). Cochrane Database Syst Rev. 2007;doi:10.1002/14651858.CD004148. 10.1002/14651858.CD004148.pub3 17443541

[14] O’Donnell A, Anderson P, Newbury-Birch D, Schulte B, Schmidt C, Reimer J, et al. The impact of brief alcohol interventions in primary healthcare: a systematic review of reviews. Alcohol Alcohol. 2014;doi:10.1093/alcalc/agt170. 10.1093/alcalc/agt170 PMC3865817 24232177

[15] Jackson R, Johnson M, Campbell F, Messina J, Guillaume L, Meier P (2009). Screening and Brief Interventions for Prevention and Early Identification of Alcohol Use Disorders in Adults and Young People.

[16] Patton R, Deluca P, Kaner E, Newbury-Birch D, Phillips T, Drummond C. Alcohol Screening and Brief Intervention for Adolescents: The How, What and Where of Reducing Alcohol Consumption and Related Harm Among Young People. Alcohol Alcohol. 2013;doi:10.1093/alcalc/agt165. 10.1093/alcalc/agt165 PMC3932830 24232178

[17] Yuma-Guerrero PJ, Lawson KA, Velasquez MM, von Sternberg K, Maxson T, Garcia N. Screening, brief intervention, and referral for alcohol use in adolescents: a systematic review. Pediatrics. 2012;doi:10.1542/peds.2011-1589. 10.1542/peds.2011-1589 22665407

[18] Calabria B, Shakeshaft AP, Havard A. A systematic and methodological review of interventions for young people experiencing alcohol-related harm. Addiction. 2011;doi:10.1111/j.1360-0443.2011.03418.x. 10.1111/j.1360-0443.2011.03418.x 21371154

[19] National Institute for Health and Care Excellence (NICE). NICE Public Health Guidance 24: Alcohol use disorders: prevention*.* National Institute for Health and Care Excellence. 2010. https://www.nice.org.uk/guidance/ph24?unlid=1595622652016227234415. Accessed 19 Apr 2017.

[20] Parkes T, Atherton I, Evans J, Gloyn S, McGhee S, Stoddart B, et al. An evaluation to assess the implementation of NHS delivered Alcohol Brief Interventions: Final Report. NHS Health Scotland. 2011; http://www.healthscotland.com/documents/5438.aspx. Accessed 15 Apr 2016

[21] Scottish Government. ABI HEAT Standard National Guidance 2012-2013. NHS Health Scotland. 2011. http://www.healthscotland.com/documents/5662.aspx. Accessed 15 Apr 2016.

[22] Scottish Government. Local Delivery Plan Standard: Alcohol Brief Interventions. National Guidance: 2015-16. 2014. Edinburgh. doi:10.1017/CBO9781107415324.004.

[23] Laird K. Scoping exercise to ascertain the current and planned range of settings for Alcohol Brief Intervention delivery in non-HEAT settings. NHS Health Scotland. 2011; http://www.healthscotland.com/documents/5603.aspx. Accessed 15 Apr 2016

[24] Ritchie J, Spencer L, Bryman A, Burgess RG (1994). Qualitative data analysis for applied policy research. Analysing Qualitative Data.

[25] Williams EC, Achtmeyer CE, Rittmueller SE, Bradley KA (2013). Local implementation of preventive alcohol care after national performance measures: Results from key-informant interviews at five VA clinics. Alcohol Clin Exp Res.

[26] Désy PM, Perhats C (2008). Alcohol screening, brief intervention, and referral in the emergency department: an implementation study. J Emerg Nurs.

[27] Johnson JA, Woychek A, Vaughan D, Seale JP (2013). Screening for at-risk alcohol use and drug use in an emergency department: integration of screening questions into electronic triage forms achieves high screening rates. Ann Emerg Med.

[28] Anderson S, Eadie DR, MacKintosh AM, Haw S (2001). Management of alcohol misuse in Scotland: the role of A&E nurses. Accid Emerg Nurs.

[29] Fitzgerald N, Youngson E, Cunningham S, Watson M, Stewart D (2015). Support for community pharmacy-based alcohol interventions: a Scottish general public survey. Public Health.

[30] Fitzgerald N, Platt L, Heywood S, McCambridge J (2015). Large-scale implementation of alcohol brief interventions in new settings in Scotland: a qualitative interview study of a national programme. BMC Public Health.

[31] Kennedy C, Finkelstein N, Hutchins E, Mahoney J (2004). Improving Screening for Alcohol Use During Pregnancy: The Massachusetts ASAP Program. Matern Child Health J.

[32] Fitzgerald N, Molloy H, MacDonald F, McCambridge J (2014). Alcohol brief interventions practice following training for multidisciplinary health and social care teams: A qualitative interview study. Drug Alcohol Rev..

[33] Damschroder L, Hagedorn H (2011). A guiding framework and approach for implementation research in substance use disorders treatment. Psychol Addict Behav.

[34] Galvani S, Hutchinson A, Dance C (2013). Identifying and Assessing Substance Use: Findings from a National Survey of Social Work and Social Care Professionals. Br J Soc Work.

[35] McCambridge J (2011). Fifty years of brief intervention effectiveness trials for heavy drinkers. Drug Alcohol Rev.

[36] Huntley JSJ, Patton R, Touquet R (2004). Attitudes towards alcohol of emergency department doctors trained in the detection of alcohol misuse. Ann R Coll Surg Engl.

[37] Doi LK. Screening and alcohol brief interventions in antenatal care : a realistic evaluation. University of Stirling. 2012. http://hdl.handle.net/1893/9513. Accessed 15 Apr 2016.

[38] Kaner E, Heather N, Brodie J, Lock C, McAvoy B (2001). Patient and practitioner characteristics predict brief alcohol intervention in primary care. Br J Gen Pract.

[39] Lock CA, Kaner EFS (2004). Implementation of brief alcohol interventions by nurses in primary care: do non-clinical factors influence practice?. Fam Pract.

[40] Hutchings D, Cassidy P, Dallolio E, Pearson P, Heather N, Kaner E (2006). Implementing screening and brief alcohol interventions in primary care: views from both sides of the consultation. Prim Health Care Res Dev.

[41] Richmond R, Kehoe L, Heather N, Wodak A, Webster I (1996). General practitioners’ promotion of healthy life styles: what patients think. Aust N Z J Public Health.

[42] Rush BR, Urbanoski KA, Allen BA (2003). Physicians’ enquiries into their patients' alcohol use: public views and recalled experiences. Addiction.

[43] Wallace PG, Haines AP. General practitioner and health promotion: what patients think. Br Med J (Clin Res Ed). 1984; doi:10.1136/bmj.289.6444.534. 10.1136/bmj.289.6444.534 PMC1442700 6432179

[44] Williams EC, Achtmeyer CE, Kivlahan DR, Greenberg D, Merrill JO, Wickizer TM (2010). Evaluation of an electronic clinical reminder to facilitate brief alcohol-counseling interventions in primary care. J Stud Alcohol Drugs.

[45] Bertholet N, Palfai T, Gaume J, Daeppen J-B, Saitz R (2013). Do Brief Alcohol Motivational Interventions Work Like We Think They Do? Alcohol Clin Exp Res.

[46] Heather N: Spreading alcohol brief interventions from health care to non-health care settings: Is it justified? Drugs Educ Prev policy, 0:000.

[47] McCambridge J, Kypri K, Elbourne D (2014). Research participation effects: a skeleton in the methodological cupboard. J Clin Epidemiol.

[48] May CR, Johnson M, Finch T Implementation, context and complexity. Implementation Science. 2016;doi: 10.1186/s13012-016-0506-3. 10.1186/s13012-016-0506-3 PMC5069794 27756414

[49] McCambridge J, Butor-Bhavsar K, Witton J, Elbourne D (2011). Can research assessments themselves cause bias in behaviour change trials? A systematic review of evidence from solomon 4-group studies. PLoS One.

[50] Solomon RL (1949). An extension of control group design. Pschol Bull.

[51] McCambridge J, Kypri K, Bendtsen P, Porter J (2013). The use of deception in public health behavioral intervention trials: a case study of three online alcohol trials. Am J Bioeth.

[52] Mdege ND, Man M-S (2011). Taylor (nee Brown) CA, Torgerson DJ: Systematic review of stepped wedge cluster randomized trials shows that design is particularly used to evaluate interventions during routine implementation. J Clin Epidemiol.

[53] Martineau F, Tyner E, Lorenc T, Petticrew M, Lock K (2013). Population-level interventions to reduce alcohol-related harm: an overview of systematic reviews. Prev Med (Baltim).

[54] McCambridge J, Strang J (2004). The efficacy of single-session motivational interviewing in reducing drug consumption and perceptions of drug-related risk and harm among young people: results from a multi-site cluster randomized trial. Addiction.

[55] Knight JR, Shrier L, Bravender T, Farrell M, Vander Bilt J, Shaffer HJ (1999). A new brief screen for adolescent substance abuse. Arch Pediatr Adolesc Med..

[56] Hodgson R, Alwyn T, John B, Thom B, Smith A (2002). The FAST alcohol screening test. Alcohol Alcohol.

[57] Saunders JB, Aasland OG, Babor TF, de la Fuente JR, Grant M (1993). Development of the Alcohol Use Disorders Identification Test (AUDIT): WHO Collaborative Project on Early Detection of Persons with Harmful Alcohol Consumption--II. Addiction.

[58] Balen R, Blyth E, Calabretto H, Fraser C (2006). Horrocks C & Manby M (2006) Involving children in health and social research: ‘Human becomings’ or ‘active beings’?. Childhood.

